# Treatment of infantile idiopathic scoliosis using a novel thoracolumbosacral orthosis: a case report

**DOI:** 10.1186/s13256-021-03168-8

**Published:** 2022-01-05

**Authors:** Jeb. McAviney, Benjamin T. Brown

**Affiliations:** 1 ScoliCare, Kirk Place, Level 5 Suite 5.08, 15 Kensington St, Kogarah, NSW 2217 Australia; 2 Faculty of Medicine, Health and Human Sciences, Macquarie University, Macquarie Park, NSW 2109 Australia

**Keywords:** Spine, Infant, Infantile scoliosis, Braces, Case reports

## Abstract

**Background:**

The recommended treatment for mild to moderate infantile idiopathic scoliosis curves involves serial casting. There are concerns, however, regarding the safety of repeated casting in very young children owing to the requirement for anesthetization during the casting process. Very little research has been conducted on the influence of bracing as an initial treatment for scoliosis in this age group. This report details the successful treatment of a large thoracic curve using a thoracolumbosacral orthosis in an infant diagnosed with infantile idiopathic scoliosis.

**Case presentation:**

The Dutch-Australian patient presented at 11 weeks of age with a 44° thoracic scoliosis and a rib vertebral angle difference of 14°. The history and physical examination failed to reveal a cause of the curvature, and a diagnosis of infantile idiopathic scoliosis was made. The patient was prescribed a thoracolumbosacral orthosis (*ScoliBrace*) to be worn on a part-time basis for a period of 8 months. At the end of the bracing program, the patient’s curve had been reduced to 7° and a rib–vertebral angle difference of 0°. A final follow-up of the patient at 2 years after the cessation of treatment revealed no evidence of scoliosis. The parents were compliant with the bracing protocol and reported that the treatment was tolerated by the infant.

**Conclusion:**

The use of an orthosis as a standalone treatment in this patient resulted in significant reduction in a large thoracic scoliosis. Based on the results witnessed in this patient, further investigation into bracing as an alternative to casting is warranted.

## Background

Infantile idiopathic scoliosis (IIS) is defined as a spinal curvature < 10° of unknown etiology occurring in an infant before the age of 2–3 years [1, 2]. The epidemiology of IIS appears to be quite variable between regions. Wynne-Davies [3] reported the incidence of scoliosis in the general population under 8 years of age to be 1.3 per 1000. Scott and Morgan [2] reported an incidence of IIS (progressive type) of 12.8% in a retrospective review of tertiary center records in England. The authors quoted an incidence of 0.25% from a similar tertiary setting in the USA, highlighting an approximate 50-fold difference in the two rates. Figures from Edinburgh, Scotland taken from presentations to a scoliosis unit between 1968 and 1982 showed declining incidence rates, from 17% down to 2%, during the review period [4]. Al-Arjani *et al.* reviewed the medical records of 192 patients seen at a spinal unit in Saudi Arabia, finding an 8% prevalence of IIS in the idiopathic scoliosis presentations. Some authors have reported that IIS is more common in males [1, 5–7]; however, a sex predilection has not been demonstrated in all studies [2]. The condition is a diagnosis of exclusion and is divided into two types: *progressive* (PIIS) and *resolving* (RIIS) [2]. As the name suggests, RIIS resolves spontaneously, whereas those with PIIS continue to worsen, often aggressively, over time.

The treatment of patients with RIIS is not deemed necessary by most authors; instead, a watch-and-wait approach is advised in this group [8]. For PIIS, the first-line conservative treatment for patients with curves measuring < 60° typically involves casting [9]. Plaster casts are molded to the intubated and anesthetized patient who is placed on a specialized casting table. A team consisting of nurses, a surgeon, and an anesthetist is required to perform the 90-minute procedure. Casts are changed every 2–4 months to accommodate patient growth. Casting is continued until the curve has been reduced to < 20° [9, 10]; after this point, an orthosis [thoracolumbosacral orthosis (TLSO)] is used to encourage the correction of any residual deformity, or as a way of stabilizing the newly straightened spine as the patient adapts to being out of the cast [8]. Casting has demonstrated efficacy for the treatment of IIS patients. There are, however, concerns that frequent anesthetization of children younger than 3 years during cast changing may impair normal brain development [11]. The use of a TLSO in IIS patients as an alternative to casting has been discouraged by some [12], but has not been extensively researched. One of the few reports by Smith *et al.* [13] highlighted only limited success in a group of 17 IIS patients treated with a TLSO. However, given that the mechanism of action underlying both bracing and casting can be similar, and that bracing in other scoliotic populations is often successful [14], further investigation is warranted.

The aim of this manuscript is to present the results of treatment for a patient with IIS using a novel bracing technique.

## Case presentation

An 11-week-old, Dutch-Australian female patient was referred to the lead author’s clinic on 5 September 2014 with suspected scoliosis. The patient’s mother had initially noticed the abnormal curvature approximately 1.5 months after birth and had taken the child to a local chiropractor. A short course of chiropractic treatment had been provided with no success, and the patient had been referred on. A standard history and physical examination were performed by the lead author. The child had been delivered naturally; however, the patient’s mother reported that the child had “gotten stuck” and support staff had to intervene. The child’s shoulder was dislocated at this time, and later relocated. The Apgar scores were normal. Both parents were of Dutch descent and the mother was 33 years old at the time of birth. The subject of this case study was the mother’s second child. There was no family history of spinal, syndromal disorder, or genetic conditions.

### Clinical findings

**Fig. 1:**
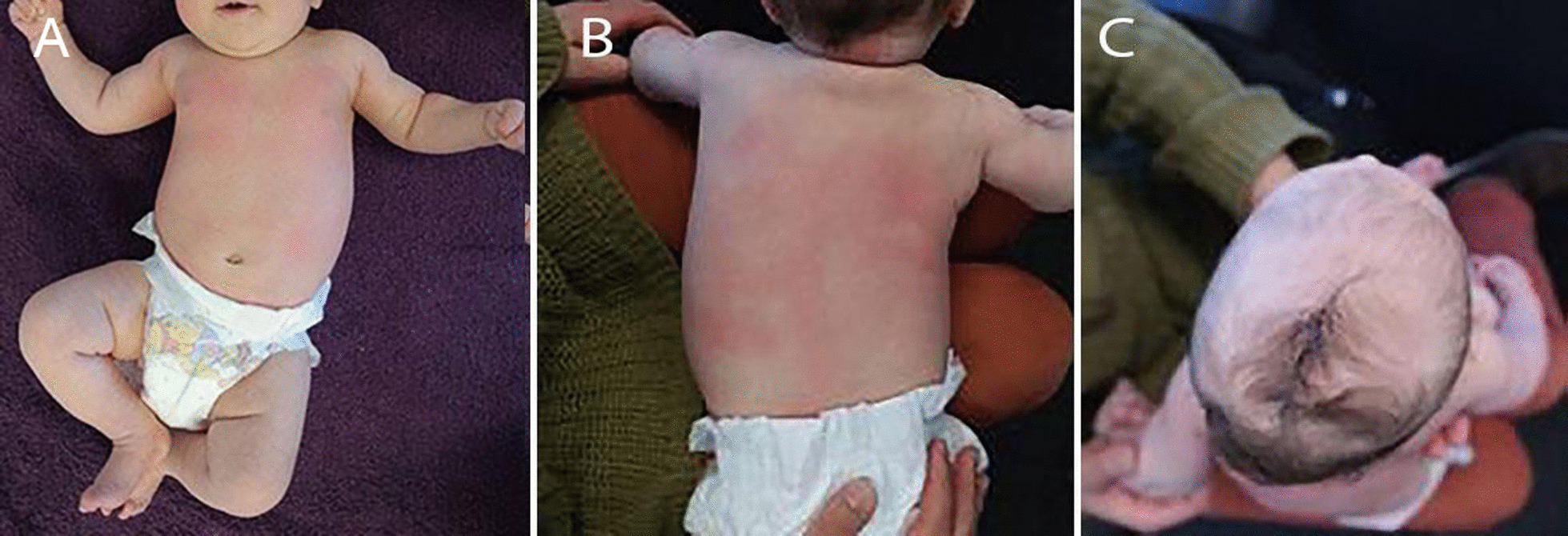
Observational findings. A scoliosis can be seen on the (**A**) anteroposterior view and (**B**) posteroanterior views. Left-sided deformational plagiocephaly was observed. **C** Superior to inferior view

**Fig. 2:**
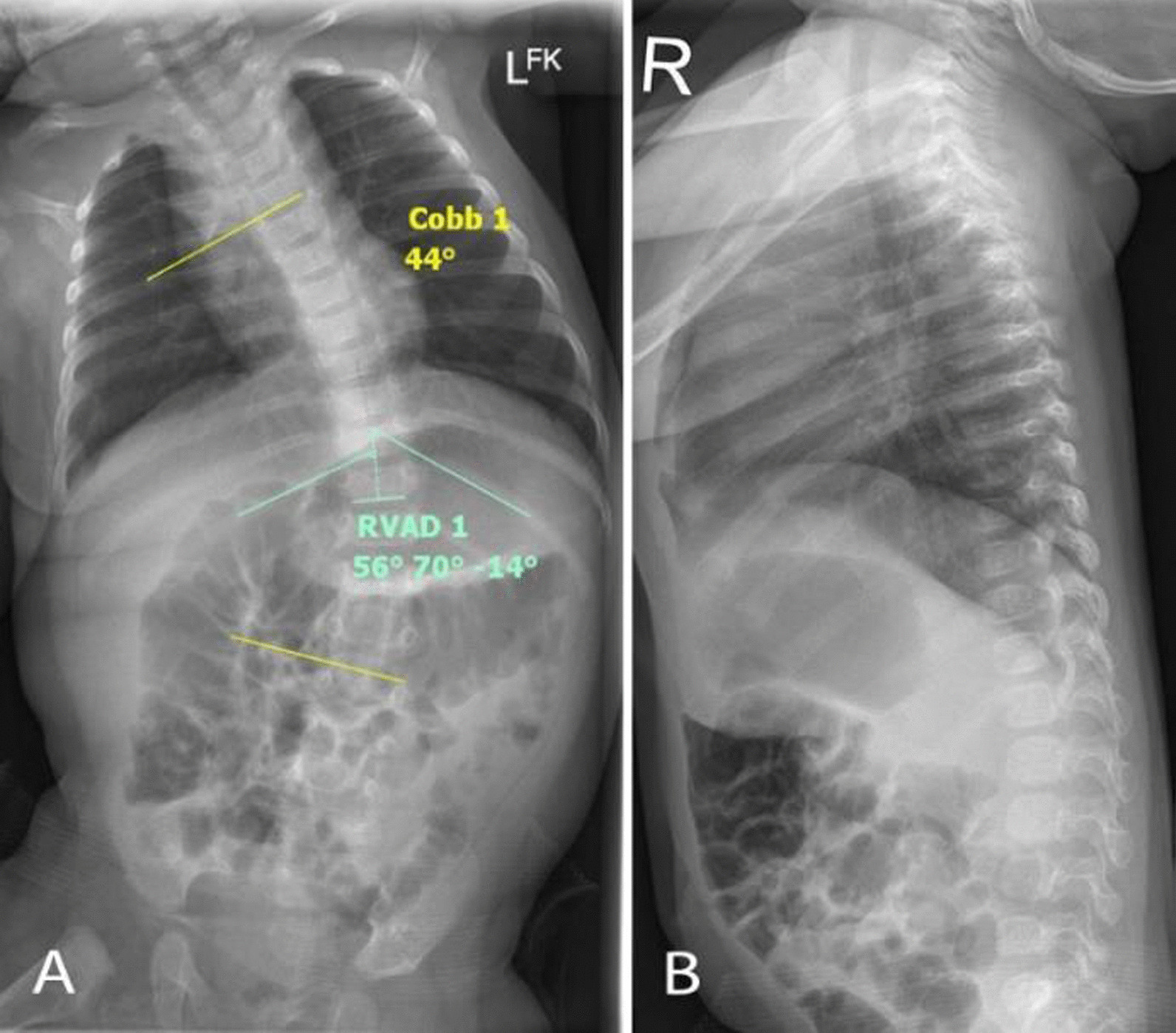
Patient x-ray findings with Cobb angle and RVAD. **A** Supine anteroposterior. **B** Right lateral decubitus

The examination performed at the time of the initial consultation revealed a single, large, left-convex, thoracic scoliosis (Fig. 1A, B) and left-sided deformational plagiocephaly (Fig. 1C). The patient demonstrated reduced left posteroanterior rotation of the cervical spine. Examination of the patient’s ears, hips, and feet was unremarkable. There was no other observable deformity or abnormality present. The curve was very rigid with little correction during side-bending and light traction. The neurological examination was within normal limits except for a slight reduction in the abdominal reflexes. Lumbar and thoracic plain films (supine anteroposterior and lateral decubitus) were ordered on the same day, which confirmed the findings from the physical examination. The single thoracic curve measured 44° (Cobb) with an apex at T11. All patient measurements described in this report were measured using the Surgimap software [15]. According to Mehta’s classification [16], the patient was in *Phase I* and had a rib–vertebral angle at the apex at the convex and concave side of the curve measuring 70° and 56°, respectively, giving a rib–vertebral angle difference (RVAD) of 14° (Fig. 2A, B). A diagnosis of IIS was made, and the patient was referred to an orthopedic surgeon for an assessment. Molded baby syndrome was listed as a differential diagnosis; however, the absence of hip contracture and ear or foot abnormalities and the direction of the cervical restriction [17, 18] made this less likely.

### Therapeutic intervention

**Fig. 3:**
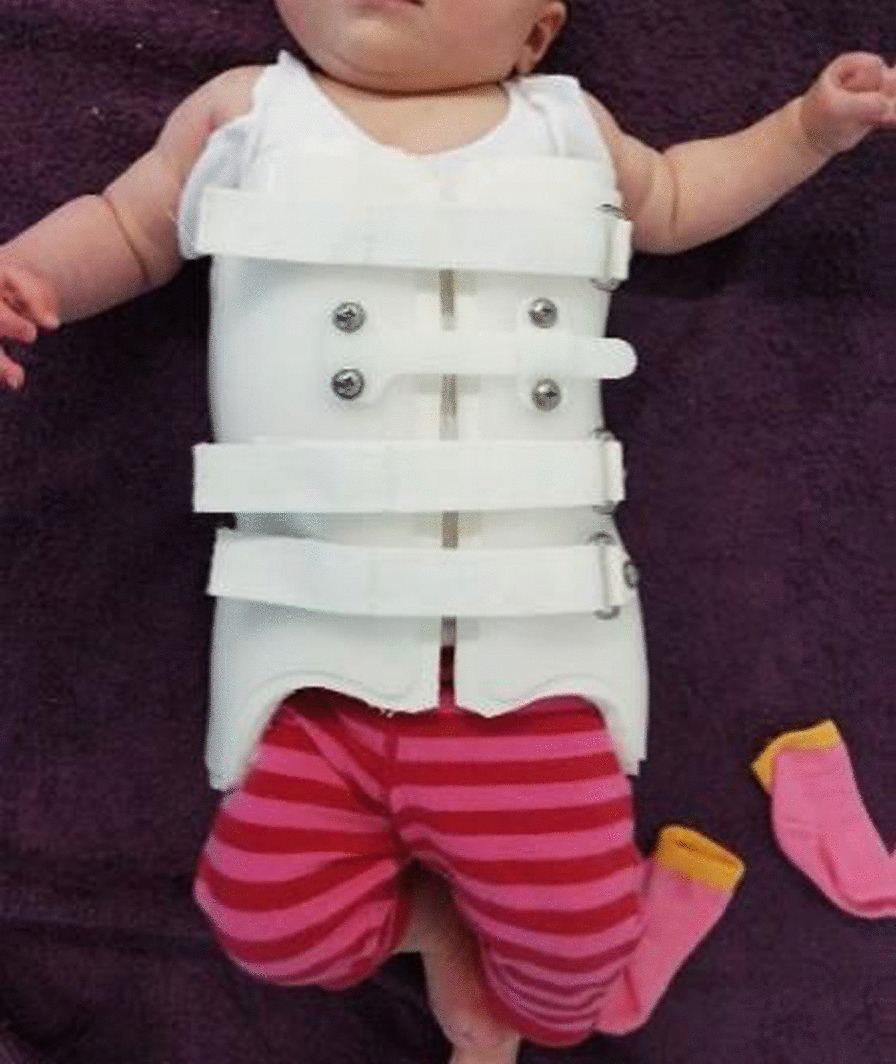
Patient wearing the rigid TLSO at the time of the brace fitting

The patient had not responded to manual therapy and was showing signs of worsening. Given that there was an absence of red flags and a substantial wait before the patient could be seen by the surgeon, the patient was prescribed a *ScoliBrace* orthosis (Sydney, Australia). The orthosis (Fig. 3) was a lightweight, rigid, over-corrective TLSO. The orthosis was customized to the patient based on three-dimensional (3D) scans and constructed using computer-aided design and manufacture software (CAD/CAM). The 3D scans are acquired using the *BraceScan* system. This system uses an infrared-laser projector that projects thousands of invisible infrared dots onto the patient. The sensor then uses a frequency-matched infrared camera to record how the pattern of light that has been projected onto the patient differs from the reflected pattern, thereby allowing for a reconstruction of the patient’s surface geometry.

The brace design was sent electronically to a factory. A 3D “negative” of the brace was created from foam, and then a single sheet of metallocene polyethylene plastic was heat molded around the negative. The orthosis was then trimmed and smoothed to match the CAD design, and then lined with *AliPlast* foam. The brace was secured to the patient’s body using three Velcro straps and a central tongue-and-groove strap.

As the brace is made in CAD, there is the opportunity to add true 3D corrective forces in the *x*, *y*, and *z* axes. For example, *y*-axis traction that is achieved with the patient under traction during the casting process can be simulated in the design of the orthosis and tightly controlled in millimeter increments. Similarly, corrective translation along the *x*-axis and corrective rotation in the *y*-axis can be added to the design. These 3D forces are traditionally very difficult to induce in a cast; hence, casting primarily relies on the *x*-axis traction force. The design approach used in the infantile *ScoliBrace* is therefore truly that of a 3D corrective approach as opposed to the traditional three-point bending (which is primarily a 2D coronal plane force) used in standard TLSO bracing.

Given the wait to see the orthopedic surgeon, the patient was closely monitored, acknowledging that bracing would be ceased if any adverse events occurred, or if any additional information came to light that would contraindicate the patient to bracing.

The patient was fitted with the brace 25 days after the initial consultation and continued with the home exercises prescribed by the chiropractor. The curve had not shown any signs of improvement during this time. The parents were advised to have the patient wear the brace daily under supervision for five 30-minute blocks in the first 2 weeks. The patient’s mother was also given instruction for donning and doffing of the brace.

### Follow-up and outcomes

After 2 weeks, brace wear was increased from 4 to 6 hours per day for another fortnight. The patient was seen by the orthopedic surgeon 46 days after the initial consultation. The surgeon ruled out any neurological lesion/s and did not request magnetic resonance imaging (MRI) or genetic screening at that time, instead recommending that a course of physiotherapy be undertaken. The patient did not proceed with the recommended physiotherapy sessions.

**Fig. 4:**
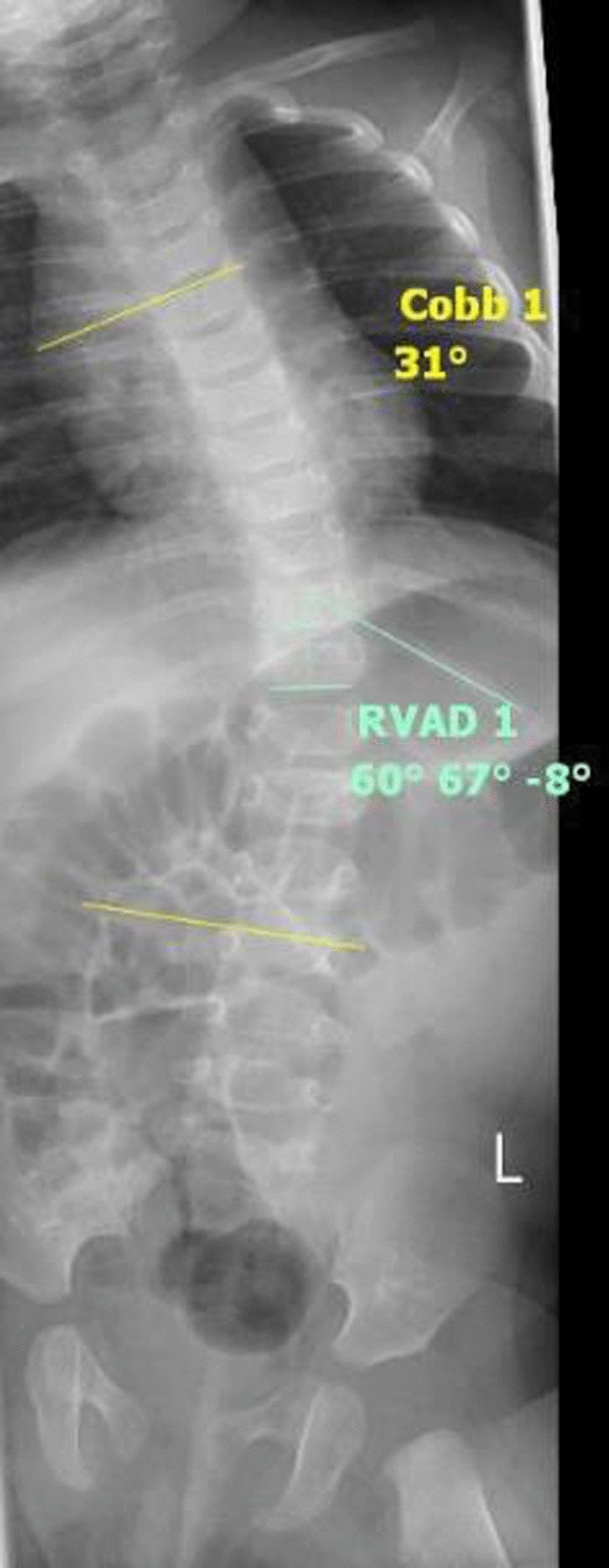
One month out-of-brace x-ray (supine anteroposterior view)

At the 1-month point in the brace treatment schedule, the curve had been reduced from 44° to 31° (RVAD 8°) (Fig. 4), and brace wear was increased to 8 hours each day for the next month. Importantly, the brace was applied under the supervision of the parents during the day and was not worn at night.

**Fig. 5:**
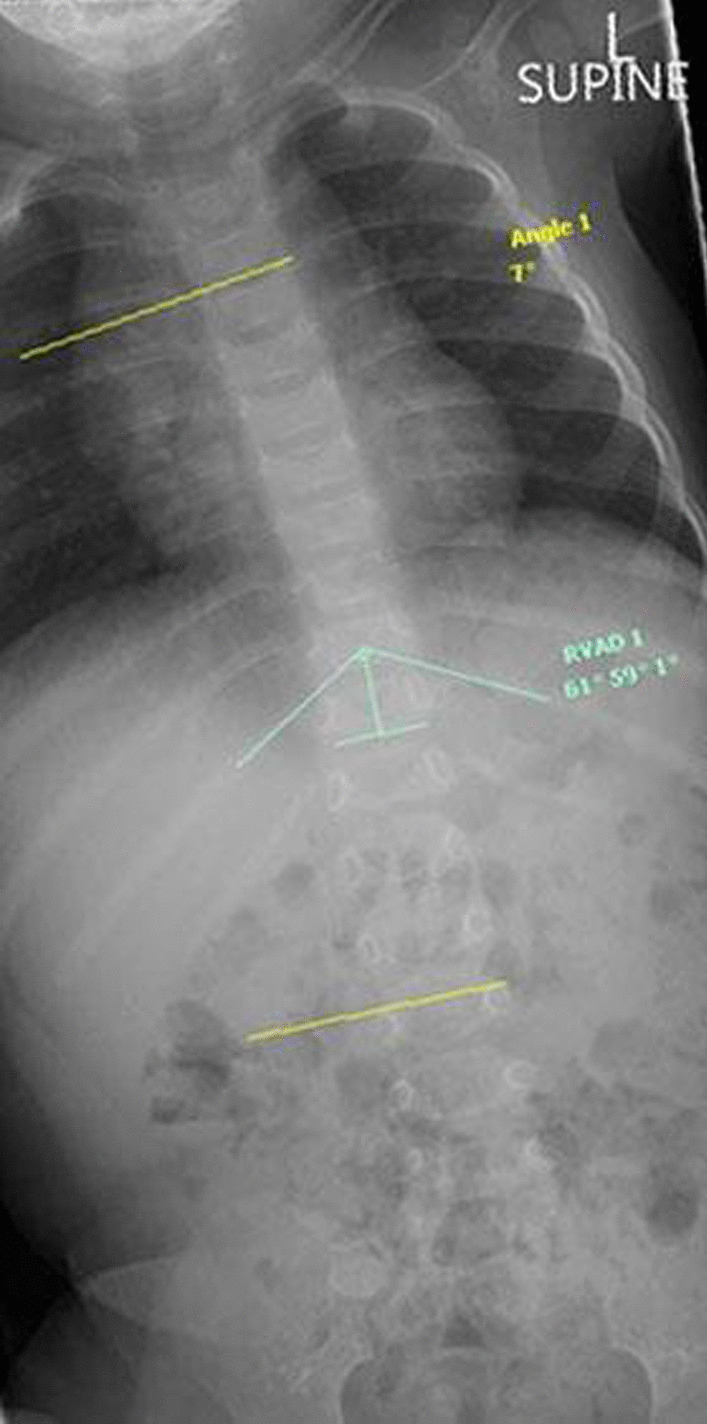
Four months out-of-brace x-ray (supine anteroposterior view). Cobb angle 7°; RVAD 1°

**Fig. 6:**
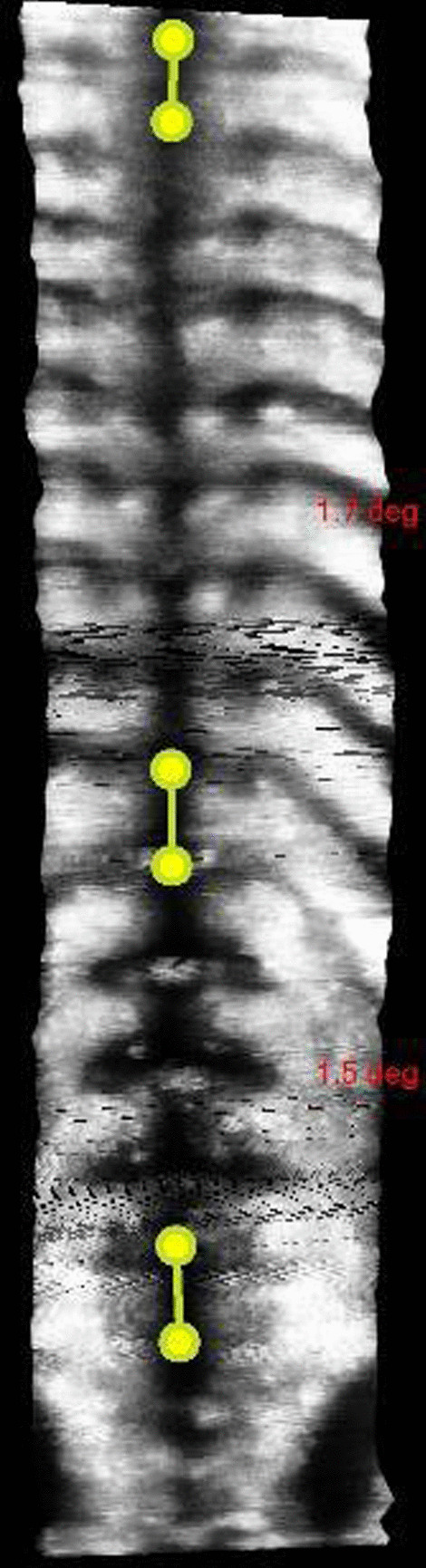
Standing 3D ultrasound image of the patient’s spine. Cobb angles (spinous process method) thoracic spine < 2°; lumbar spine < 2°

**Fig. 7:**
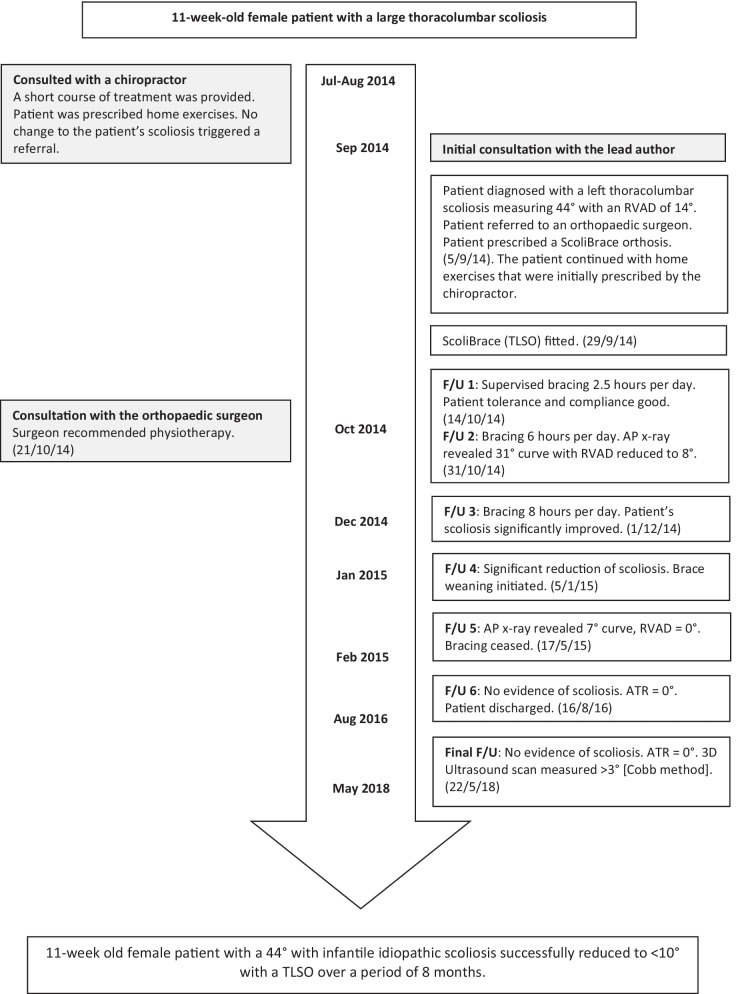
Case timeline

After 3 months wearing the brace, the patient’s scoliosis had reduced significantly. Brace weaning was commenced, with the patient reducing the brace wear from 8 to 4 hours per day for the next 4 months. Plain films taken 4 months after the brace fitting revealed that the patient’s scoliosis had reduced from 44° to 7° and the RVAD had reduced from 14° to 1° (Fig. 5). The patient was followed up again 23 months after the initial consultation. There was no evidence of scoliosis or rib humping at this time, and the patient’s angle of trunk rotation (ATR) was < 5°. A final review was performed 2 years after the cessation of brace wear. The Adam’s forward bend test was negative, and ATR measurements were within normal limits. A standing 3D ultrasound image of the patient’s spine was taken using the *Scolioscan* (Telefield Medical Imaging Ltd, Hong Kong), which did not reveal any abnormal curvature of the spine using the spinous process method of Cobb angle evaluation [19, 20] (Fig. 6). A flow chart depicting the timeline of management for this case is provided in Fig. 7.

Compliance with the bracing was good and generally well tolerated by the patient and her parents. Minor modifications were made to the orthosis at several points along the treatment schedule. These modifications involved removing padding, cutting away plastic, and flaring out sections of the brace around the arms and legs to accommodate the rapid growth of the patient during this period. In terms of adverse reactions, the mother stated that the child’s arm would occasionally change color if the child was being carried in an upright position while in the brace. This was most likely due to compression of the neurovascular structures in the axillary region by the uppermost edge of the orthosis. This is a possible side effect if the orthosis is worn too loosely. In terms of comfort, the mother did report that the child would cry at times when wearing the brace, which made adherence to the treatment a little more challenging.

## Discussion

The authors have presented a case report detailing the successful treatment of an 11-week-old female patient with a large IIS curve using a *ScoliBrace* TLSO. The patient presented with a scoliosis that was reduced from 44° to 7° over a period of 8 months. Adam’s forward bend testing, scoliometer readings, and spinal ultrasound taken at the most recent follow-up consultation (2 years after the cessation of treatment) suggest a complete resolution of the patient’s scoliosis. Rather than casting the patient, a 3D scanning system was used to obtain a 3D model of the patient’s body for the purposes of design and manufacture of an orthosis. The use of CAD in the design gave the opportunity to not only introduce *y*-axis traction forces to the spine similar to casting, but also allowed the application of corrective forces in the transverse and coronal planes. Traditional TLSO braces rarely address the true 3D nature of a scoliosis and tend to treat the coronal plane deformity only.

Moreover, the fact that the brace could be donned and doffed easily not only made the treatment highly functional/flexible for the parents of such a young child, but also made graduated treatment and weaning possible. An orthosis such as the one used in this case can be quickly removed to accommodate flare-ups of comorbid medical conditions, and is more resistant to soiling when compared with a plaster cast.

There are a few limitations to this case study, namely uncertainty regarding a precise diagnosis for the patient. Based on the RVAD and rib phase information obtained from the initial radiographs, the patient would have likely been placed at a low risk for curve progression and assumed to be a case of RIIS [16]. However, because of the the magnitude of the curve, the absence of pretreatment radiographs detailing the rate of curve progression in this patient, and the fact it had not resolved with manual therapy and exercise, it was unclear which type of IIS the patient presented with. Moreover, Mehta [16] acknowledged that not all patients who present *Phase I* with RVAD measurements < 20° experience a resolution of their scoliosis, and not all curves observed in this subgroup adhere to this classification system. However, running with the assumption that this was a case of RIIS, Scott and Morgan [2] state that the resolution of scoliosis in RIIS patients takes between 2 and 5 years, with a mean of 2.5 years. If this was a RIIS case, then the treatment regimen described in this report has possibly reduced the resolution time. However, Lloyd-Roberts and Pilcher [21] state that the vast majority (92%) of scoliosis witnessed in children under the age of 1 year will resolve spontaneously after approximately 12 months. Based on the uncertainty in the diagnosis, the initial Cobb angle, and the knowledge that curves in PIIS can advance rapidly in patients < 2 years of age owing to the very high growth velocity during this period, the decision was made to intervene.

With reference to etiology, it is not known what role the dislocation and subsequent relocation of the patient’s shoulder, at the time of the delivery, had on the development of the patient’s scoliosis. Unfortunately, there is a dearth of literature on this topic to aid in our interpretation of this aspect of the case.

Casting is currently viewed as the treatment of choice for patients with mild to moderate PIIS. There are, however, aspects of this intervention that are undesirable, namely: the cost, the high technical staffing and equipment requirements, and the repeated exposure of the patient’s developing brain to anesthetizing agents during the casting process. Furthermore, it is difficult to predict which patients will respond favorably to casting [22]. The patient in this report has obtained a positive result and has potentially been spared the inconvenience and potential harms associated with casting treatment.

## Conclusion

The use of a uniquely designed orthosis in this patient has resulted in a significant reduction of a 44° thoracic scoliosis. The use of orthoses in general as a first-line treatment option for IIS patients is not well regarded in the literature. There is, however, a paucity of evidence to justify the disparagement of this intervention. With advances in the technology associated with bracing and the harms associated with casting treatment, further investigation into this specific style of treatment is warranted.

### Parent’s perspective

The bracing regimens was generally well tolerated by the patient and her parents. The patient’s mother reported that her daughter did cry at times while being in the brace; however, compliance was very good, suggesting that the treatment regimen was suitable for the patient and could be fit into the life/schedule of the parents. There was one adverse reaction reported (change in the color of the arm from pressure), but this could be easily avoided with relevant advice and instruction regarding brace wear. The patient’s mother reported that her child had achieved all the normal developmental milestones and was very strong, healthy, and straight. The patient’s mother stated that they would not do anything differently if placed in the same situation again. After the final follow-up, the patient’s parents were advised to monitor for signs and symptoms of scoliosis and to schedule a follow-up if any concerning features were observed.

### Informed consent

The patient’s guardian (mother) provided informed consent for her daughter’s case details and de-identified personal information and images to be used in this manuscript.

## Data Availability

Not applicable.

## Abbreviations

2D: Two-dimensional
3D: Three-dimensional
ATR: Angle of trunk rotation
CAD: Computer-aided design
CAM: Computer-aided manufacture
IIS: Infantile idiopathic scoliosis
LAT: Lateral
mPE: Metallocene polyethylene
PIIS: Progressive infantile idiopathic scoliosis
RIIS: Resolving infantile idiopathic scoliosis
RVAD: Rib–vertebral angle difference
TLSO: Thoracolumbosacral orthosis
